# Gender Differences in Hemocyte Immune Parameters of Hong Kong Oyster *Crassostrea hongkongensis* During Immune Stress

**DOI:** 10.3389/fimmu.2021.659469

**Published:** 2021-03-31

**Authors:** Jie Lu, Yanyan Shi, Tuo Yao, Changming Bai, Jingzhe Jiang, Lingtong Ye

**Affiliations:** ^1^ Key Laboratory of South China Sea Fishery Resources Exploitation and Utilization, Ministry of Agriculture and Rural Affairs, South China Sea Fisheries Research Institute, Chinese Academy of Fishery Sciences, Guangzhou, China; ^2^ Department of Chemical and Biochemical Engineering, College of Chemistry and Chemical Engineering, Xiamen University, Xiamen, China; ^3^ Key Laboratory of Maricultural Organism Disease Control, Ministry of Agriculture and Rural Affairs, Qingdao Key Laboratory of Mariculture Epidemiology and Biosecurity, Yellow Sea Fisheries Research Institute, Chinese Academy of Fishery Sciences, Qingdao, China; ^4^ Key Laboratory of Aquatic Product Processing, Ministry of Agriculture and Rural Affairs, South China Sea Fisheries Research Institute, Chinese Academy of Fishery Sciences, Guangzhou, China

**Keywords:** gender-based difference, cellular immunity, hemocyte subpopulations, *Crassostrea hongkongensis*, immune stimulation

## Abstract

Gender differences in individual immune responses to external stimuli have been elucidated in many invertebrates. However, it is unclear if gender differences do exist in the Hong Kong oyster *Crassostrea hongkongensis*, one of the most valuable marine species cultivated along the coast of South China. To clarify this, we stimulated post-spawning adult *C. hongkongensis* with *Vibrio harveyi* and lipopolysaccharide (LPS). Gender-based differences in some essential functional parameters of hemocytes were studied *via* flow cytometry. Obvious gender-, subpopulation-, and immune-specific alterations were found in the hemocyte immune parameters of *C. hongkongensis*. Three hemocyte subpopulations were identified: granulocytes, semi-granulocytes, and agranulocytes. Granulocytes, the chief phagocytes and major producers of esterase, reactive oxygen species, and nitric oxide, were the main immunocompetent hemocytes. Immune parameter alterations were notable in the accumulation of granulocyte esterase activities, lysosomal masses, nitric oxide levels, and granulocyte numbers in male oysters. These results suggest that post-spawning-phase male oysters possess a more powerful immune response than females. Gender and subpopulation differences in bivalve immune parameters should be considered in the future analysis of immune parameters when studying the impact of pathogenic or environmental factors.

## Introduction

Gender-specific differences in hemocyte immuno-competence have been reported in several aquatic invertebrates (1, 2). For example, in the sea urchin *Paracentrotus lividus*, females possess more immunocytes, consisting of phagocytes, uncolored spherulocytes, and the coelomocyte lysate, than males (3). Studies on the immune system of the clam (*Ruditapes philippinarum*) showed that, during the pre-spawning period, females have more active hemocytes than males (4). A higher phagocytic index was observed in female triploids compared with male Pacific oysters (*Crassostrea gigas*) (5). In contrast, males of the sea cucumber *Apostichopus japonicus* have a stronger antioxidant ability and more effective complement system than females after spawning (6). These studies suggest that gender-based differences in immune function and disease susceptibility are a common feature of aquatic invertebrates.

Many studies of bivalves have reported the impacts of external factors, such as pathogenic bacteria (7), salinity (8), temperature (9), and pollutants (10, 11), on hemocyte immune parameters. However, few reported investigations have examined gender-related differences in immune parameters in response to environmental factors. The phagocytic activity of female blue mussels, *Mytilus edulis*, showed a higher sensitivity to mercury than that of the males (12). Female *C. corteziensis* oysters were found to be more susceptible than males to thermic, mechanical, and mechanical-thermic stress conditions (13). Apoptosis, mortality, and oxidative stress in male New Zealand Greenshell™ mussels (*Perna canaliculus*) were observed to increase after exposure to *Vibrio* sp. DO1 (1). These studies have provided evidence of gender-based differences in some immune parameters of hemocytes toward external factors. However, bivalve hemocytes are composed of multiple functional heterogeneous cell types, and the various cell types have different functions (14). Therefore, gender-related differences in the immune parameters of hemocyte subpopulations should be investigated.

The hemocytes of bivalves can typically be separated into several subpopulations based on their morphological and cytochemical features, such as cell size, granularity, and nucleus-cytoplasm (N:C) ratio (14). Many studies have led to the characterization of the hemocyte subpopulations of different bivalves, such as green-lipped mussel (*Perna canaliculus*) (15), horse mussel (*Modiolus kurilensis*) (16), and pearl oyster (*Pteria hirundo*) (17). For example, circulating hemocytes of eastern oysters (*C. virginica*) were classified as agranulocytes, intermediate hemocytes, granulocytes, and small granulocytes (18). *C. gigas* hemocytes were grouped into three morphologically different subpopulations that included agranulocytes, semi-granulocytes, and granulocytes (19). Although different hemocyte populations have been reported for many bivalves, classifying the hemocyte morphologies in individual species is necessary, as not all bivalves have the same types and proportions of hemocytes (20, 21). Additionally, differences in hemocyte subpopulations may be important causative factors in the above-mentioned gender-based differences in the immune parameters of hemocytes. However, few reports are available on gender-related differences in the immune responses of subpopulations after immune stimulation.

In the present study, we aimed to investigate gender-specific differences in the immunological responses of different oyster hemocyte subpopulations following exposure to lipopolysaccharide (LPS) and *Vibrio harveyi*. The hemocyte subpopulations in the Hong Kong oyster *C. hongkongensis* were separated by flow cytometry based on their morphological features. Molecular probes were then used to characterize the cells’ corresponding immune functions.

## Materials and Methods

### Oyster and Hemocyte Collection

Healthy post-spawning adults of *C. hongkongensis* (shell height 11.23 ± 0.06 cm) were collected in July 2020 from a commercial farm in Taishan, Jiangmen, Guangdong Province, China. The oysters were maintained in aerated sand-filtered seawater at a salinity of 20 ± 1 psu and temperature of 23-25°C, and fed twice daily with *Isochrysis galbana* and *Chaetoceros muelleri* for 7 days.

*Vibrio harveyi* was cultured in 2216 broth at 28°C for 14 h and harvested by centrifugation (5000 × *g*, 10 min). After washing twice with aseptic seawater, *V. harveyi* was resuspended in aseptic seawater at a final concentration of approximately 1 × 10^7^ CFU/mL. LPS (from *Escherichia coli* O111: B4, Sigma) was dissolved in aseptic seawater to a concentration of 0.5 mg/mL. We randomly divided 180 oysters into three groups, and each received injections of 100 μL LPS solution (LPS group), *V. harveyi* suspension (*V. harveyi* group), or aseptic seawater (control group) into the adductor muscle. Each group contained three replicates, with 20 oysters per replicate.

The hemolymph was sampled from the posterior adductor muscle of *C. hongkongensis* at 24 h post-injection using a 5-mL syringe fitted with a 22-G needle and mixed immediately with an equal volume of modified Alsever’s solution (glucose 20.8 g/L, sodium chloride 13.5 g/L, sodium citrate 8.0 g/L, EDTA-Na_2_ 4.28 g/L, 600 mOsm/kg, 0.22 μm filtered), then centrifuged at 4°C, 500 × *g* for 10 min. The hemocytes pellets were resuspended to 1.5-2 × 10^6^ cells/mL in modified L15 medium (Leibovitz’s L15 medium with 4.42 g/L NaCl, 3.9 g/L MgCl_2_, 1 g/L MgSO_4_, 0.6 g/L CaCl_2_, 0.54 g/L KCl, streptomycin 100 mg/mL, penicillin 100 IU/mL, 600 mOsm/kg, 0.22 μm filtered) for later analysis. To reduce individual variation, the hemocytes from three individuals per group were pooled into one sample, and at least five male and five female replicates were used in the following assays. Oyster sex was judged by visually inspecting the males and females releasing gametes. The hemocyte concentration in the hemolymph was evaluated using manual counting methods with a Neubauer chamber.

### Subpopulations Analysis of Hemocytes

The histological characterization of hemocytes was performed under light microscopy following Wright-Giemsa staining (22). Stained slides were observed using a light microscope (Leica DM2000, Leica, Heerbrugg, Switzerland), and hemocyte subpopulations were characterized according to their morphological features.

Flow cytometric analyses of the hemocytes subpopulations were conducted with a FACS Arial II flow cytometer (Becton, Dickinson and Company). Briefly, 200 μL of hemocyte suspension was stained with SYBR-Green I (10× final concentration, Invitrogen, Life Technologies) in the dark for 1 h at 25°C. The fluorescence emissions were measured in the FL1 channel (530 nm). Hemocyte subtypes were distinguished using the SYBR Green positive cell density-plot according to their morphological parameters, side scatter (SSC) for internal granularity, and forward scatter (FSC) for relative size.

### Measurement of Immune Parameters by Flow Cytometry

The hemocyte parameters were analyzed using FACS Arial II flow cytometry. A total of 10,000 events were acquired for each sample. The data were displayed as cell cryptograms indicating the relative size, internal granularity, and fluorescence channels corresponding to the fluorescent markers used. The fluorescence frequency distribution histogram of each hemocyte subpopulation was then obtained. The fluorescence recorded depended on the monitored immunological parameters: hemocyte late apoptosis or necrosis was measured in the propidium iodide (PI) channel (610/20 nm), and the others were evaluated in the FITC channel (530/30 nm). The data were analyzed using FlowJo v10.3 software (FlowJo LLC, Ashland, OR). All analyses were completed within 2 hours.

Apoptosis and necrosis in hemocytes were tested with a commercial detection kit using Annexin V-FITC and PI according to the optimized manufacturer’s instructions (Beyotime Biotechnology, China). Briefly, 100·µL of hemocyte suspension (0.5-1 × 10^6^ cells/mL in annexin V-FITC binding buffer adjusted with NaCl to be isotonic to the oysters’ environments) was incubated with 5·µL Annexin V and 10·µL PI solutions. After a 15 min incubation at 25°C in the dark, the cell solutions were diluted 1:4 with binding buffer. Early apoptosis-associated fluorescence (FITC) and late apoptosis or necrosis-associated fluorescence (PI) were measured by flow cytometry. The bivariate analysis allowed the discrimination of viable (FITC−/PI−), early apoptotic (FITC+), and late apoptotic or necrotic hemocytes (FITC+/PI+).

The phagocytic activity was measured using 1-µm diameter yellow-green fluorescent polystyrene beads (Fluoresbrite, PolyScience 17154). The hemocytes were incubated in M-L15 in the dark for 1 h at 25°C at a 1:100 hemocyte-bead ratio before flow cytometry analysis. The phagocytic activity of each hemocyte subpopulation was expressed as the percentage that engulfed at least three fluorescent beads.

The mitochondrial mass, lysosomal mass, non-specific esterase activity, reactive oxygen species (ROS) level, nitric oxide (NO) level, and intracellular calcium concentration were measured using commercialized probes and chemical compounds (Beyotime Biotechnology, China) by following the manufacturer’s instructions. Briefly, 200 μL of hemocyte suspension was mixed with the corresponding probes, then incubated at 25°C in the dark before processing with flow cytometry. The final concentration and incubation time of the probes are listed in **Table 1**. The parameters in each hemocyte subpopulation were expressed as the mean fluorescence intensity (MFI) in arbitrary units (A.U.).

**Table 1:** Final concentration and incubation time of probes used in this study.

Immune parameters	Fluorescent probe	Final concentration	Incubation time
Mitochondrial mass	Mito-Tracker Green	100 nmol/L	15 min
Lysosomal mass	Lyso-Tracker Green DND-26	75 nmol/L	45 min
Non-specific esterase activity	Fluorescein diacetate	5 μmol/L	30 min
ROS	DCFH-DA	10 mmol/L	20 min
NO	DAF-FM DA	5 mmol/L	20 min
Calcium concentration	Flou-4 AM	2 mmol/L	20 min

### Statistical Analysis

The data were first tested for normality using the Shapiro-Wilk’s test and for homogeneity of variance using Levene’s test. Percentage data were arcsine-transformed, and other data were log10 transformed. Principal component analysis (PCA) was used to characterize the relationships among the immune function variables. Two-way MANOVA was used to test for the gender, immune stimulation, and interaction effects on all measured parameters, and Pillai’s trace was used to assess significance. Two-way ANOVA was then used to test for gender, immune stimulation, and interaction effects on each measured parameter. We used Tukey’s multiple comparisons test for *post hoc* analysis to compare individual means. Spearman’s correlation analysis was used to assess the relationship among the immunological parameters with the corrplot (23) and corrr (24) packages in R. Data are presented as the mean ± standard deviation (SD), and *p* < 0.05 was used to determine significance.

## Results

### Microscopic and Flow Cytometric Characteristics of the Hemocytes

**Figure 1:**
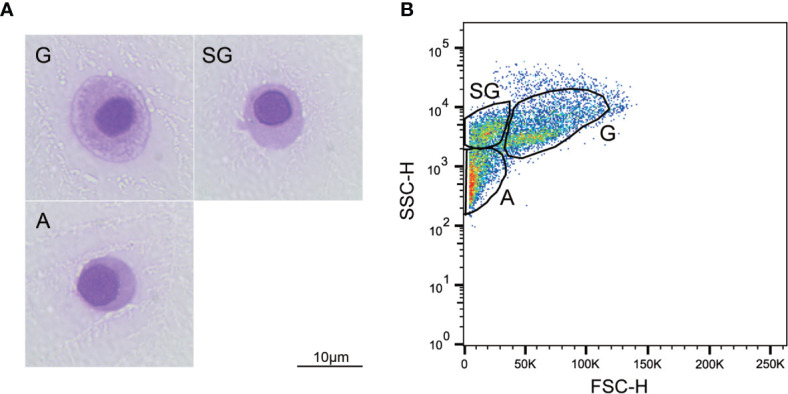
Morphological characterization of *C. hongkongensis* hemocytes. **(A)** Light micrographs of different hemocyte subpopulations after Wright-Giemsa staining. **(B)** Flow cytometric dot plot of size (FSC) against internal complexity (SSC) of hemocyte subpopulations of a representative sample. G, granulocytes; SG, semi-granulocytes; A, agranulocytes.

The cytological observations outlined in **Figure 1A** show that three subtypes of hemocytes, agranulocytes (A), semi-granulocytes (SG), and granulocytes (G), were identified in the Hong Kong oyster *C. hongkongensis* based on size and internal complexity on Wright-Giemsa staining. The cells were further classified using the cell density plot, which represents the relative cell size (FSC-H) and internal complexity (SSC-H) from the flow cytometry analysis (**Figure 1B**). Specifically, G, the largest cell subpopulation, was characterized by cytoplasm with many large granules and a relatively small N:C ratio; whereas A represented the smallest and the least complex cells with no granules in the cytoplasm and the largest N:C ratio. SG were identified as median types between agranulocytes and granulocytes. No significant differences were detected between males and females with regards to the size and complexity of the hemocytes.

### Functional Characterization of Hemocytes Subpopulations

#### Multivariate Data Analyses of All Hemocyte Subpopulations

**Figure 2:**
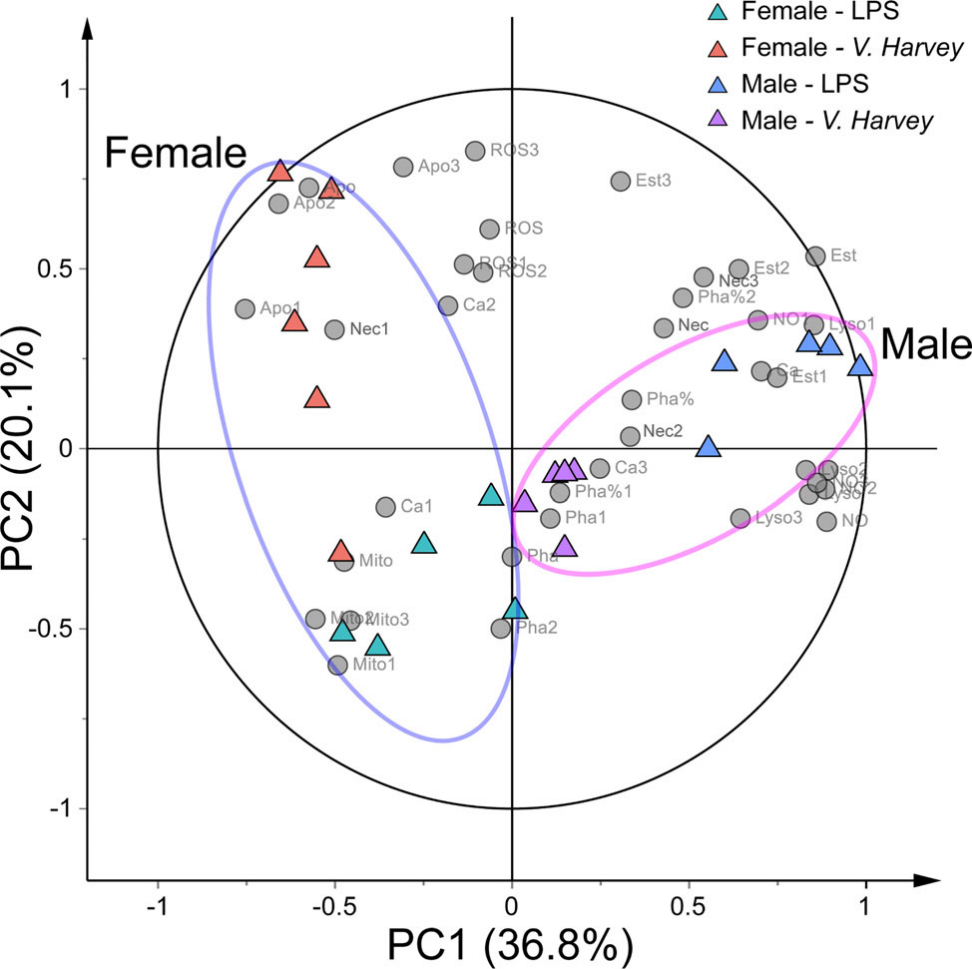
PCA biplot showing the relationships among all immunological parameters and four exposure groups (LPS-infected males, LPS-infected females, *V. harveyi*-infected males, and V. harveyi-infected females) for all hemocyte subpopulations. 1, granulocytes; 2, semi-granulocytes; 3, agranulocytes. Mito, mitochondrial mass; NO, nitric oxideNO level; Ca, calcium content, Pha, phagocytic ratio; Est, esterase activity; ROS, ROS level; Lyso, lysosome mass; Apo, early apoptotic ratio; Nec, late apoptotic or necrotic ratio.

We observed strong immune stimulation, gender, and interaction effects on all measured parameters (MANOVA, Pillai’s trace = 4.582, *F*_22,32_ = 1.518, *p* < 0.001; Pillai’s trace = 0.730, *F*_11,15_ = 3.692, *p* = 0.01; and Pillai’s trace = 1.628, *F*_22,32_ = 1.980, *p* < 0.001, respectively). Additionally, PCA was performed on the immunological parameters to identify intrinsic immunological trends and the differential immunological parameters responsible for stimulation. PCA showed that 56.9% of the total variance was explained by two principal components (**Figure 2**). PC1 represented 36.8% of the total variance, indicating a significant separation between males and females. The characteristics of the hemocyte functions associated with males were higher NO levels, lysosome mass, and esterase activities and late apoptotic or necrotic ratios coupled with lower early apoptotic ratios. Moreover, there was a clear separation between the four stimulation groups on PCA.

#### Composition Changes in Hemocytes After Stimulations

**Figure 3:**
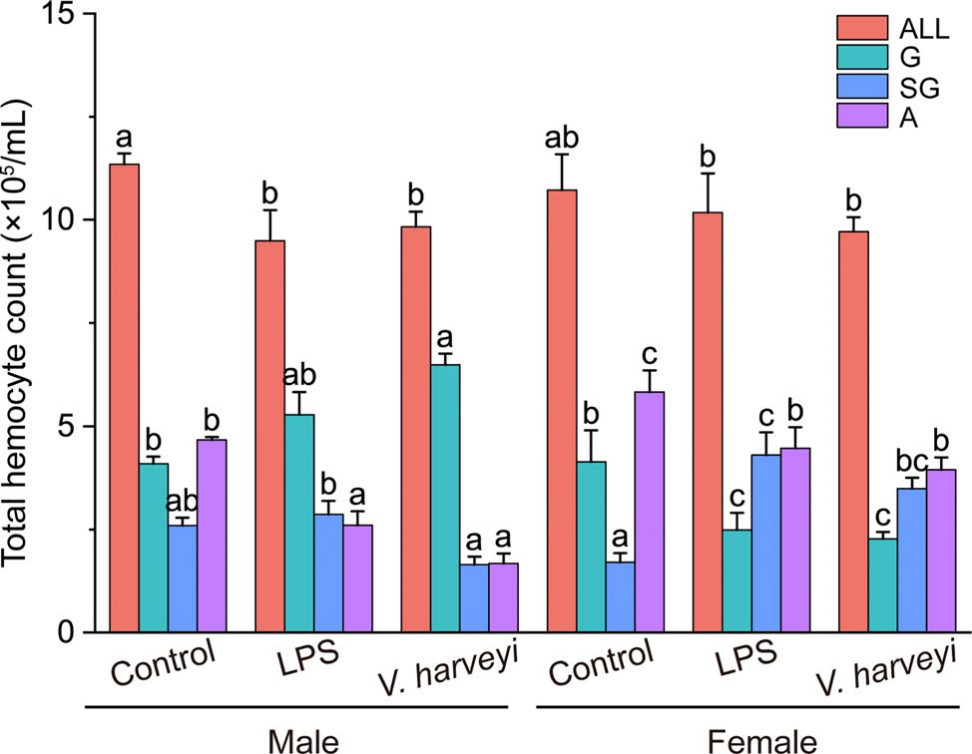
Number of all hemocyte (ALL), granulocytes (G), semi-granulocytes (SG), and agranulocytes (A) of female and male oysters after LPS, *V. harveyi*, or control stimulation. The means denoted by different letters at each fixed hemocyte subpopulation are significantly different among different treatments (*p* < 0.05).

The hemocyte concentration in *C. hongkongensis* under the control conditions was 1.12 ± 1.1 × 10^6^ cells/mL. The total hemocyte count (THC) did not vary statistically between the genders under each fixed condition but was reduced by two immune stimulations. Additionally, the sizes of three hemocyte subpopulations were significantly affected by gender, immune stimulation, and their interactions, at most time points in the experiment (**Table S1**). In male oysters, granulocyte and agranulocyte numbers significantly increased and decreased, respectively, after the two immune stimulation types (**Figure 3**); however, in females, the number of semi-granulocytes significantly increased, whereas granulocytes and agranulocytes decreased, after the two immune stimulations (**Figure 3**).

#### Annexin V-FITC/PI Assay

**Figure 4:**
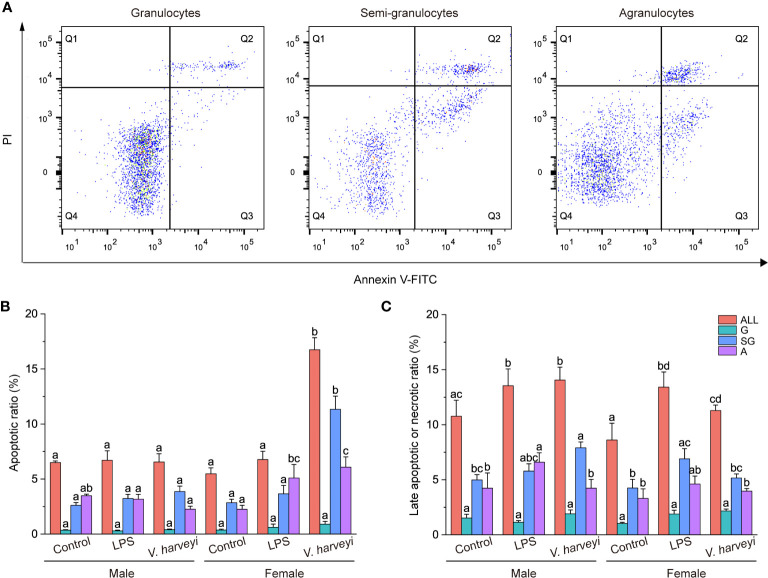
Results of the annexin V-FITC and propidium iodide (PI) assay. **(A)** Representative scatter diagrams of three hemocyte subpopulations from *C. hongkongensis*. **(B)** Early apoptotic hemocyte ratios of different hemocyte subpopulations. **(C)** Late apoptotic or necrotic hemocyte ratios of different hemocyte subpopulations. The means denoted by different letters for each fixed hemocyte subpopulation are significantly different among different treatments (*p* < 0.05). G, granulocytes; SG, semi-granulocytes; A, agranulocytes.

**Figure 4A** shows representative Annexin V-FITC vs PI scatter diagrams for the different hemocyte subpopulations, with quadrant gates showing four populations. Most granulocytes were viable and non-apoptotic. Data from the four populations were further plotted in **Figures 4B, C**, which showed that both apoptotic and necrotic ratios were significantly higher in semi-granulocytes and agranulocytes than in granulocytes.

The early apoptotic ratios of total hemocytes, semi-granulocytes, and agranulocytes were significantly affected by immune stimulation, gender, and their interactions, at most time points (**Table S1**). Immune stimulation had no effect on the early apoptotic ratios of hemocyte subpopulations in male oysters but affected female oysters. Challenging the oysters with *V. harveyi* significantly increased the early apoptotic ratios of the semi-granulocytes and agranulocytes. Both LPS and *V. harveyi* stimulations significantly increased the late apoptotic or necrotic ratios of all hemocytes.

#### Phagocytic Activities of Hemocyte Subpopulations

**Figure 5:**
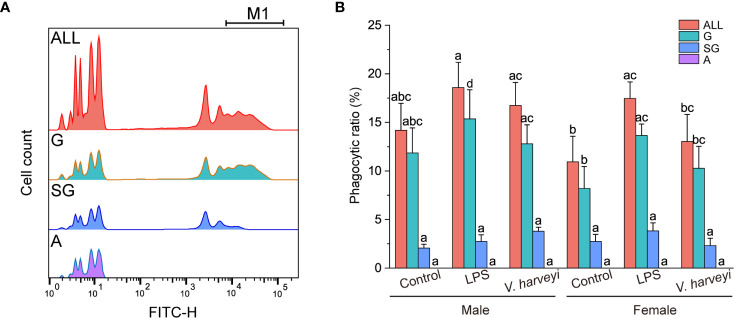
Phagocytic capability of each hemocyte subpopulation. **(A)** Histogram of fluorescence representing phagocytic activity recorded in different hemocyte subpopulations: M1, hemocytes that engulfed three or more fluorospheres. **(B)** Phagocytic ratios of different subpopulations after stimulation. The means denoted by different letters for each fixed hemocyte subpopulation are significantly different among the different stimulation types (*p* < 0.05). G, granulocytes; SG, semi-granulocytes; A, agranulocytes.

Flow cytometry and fluorescent microspheres were used to detect the phagocytic activities of the different subpopulations. Both granulocytes and semi-granulocytes showed phagocytic capacities, whereas agranulocytes did not (**Figure 5A**). The percentage phagocytosis of granulocytes was significantly higher (*p* < 0.001) than that of semi-granulocytes (**Figure 5B**). The phagocytic ratios of total hemocytes and granulocytes were significantly affected by interactions between immune stimulation and gender during the experiment (**Table S1**), and the phagocytic indexes of granulocytes showed a significant increase after LPS stimulation (**Figure 5B**).

#### Six Immunological Parameters of Hemocyte Subpopulations

**Figure 6:**
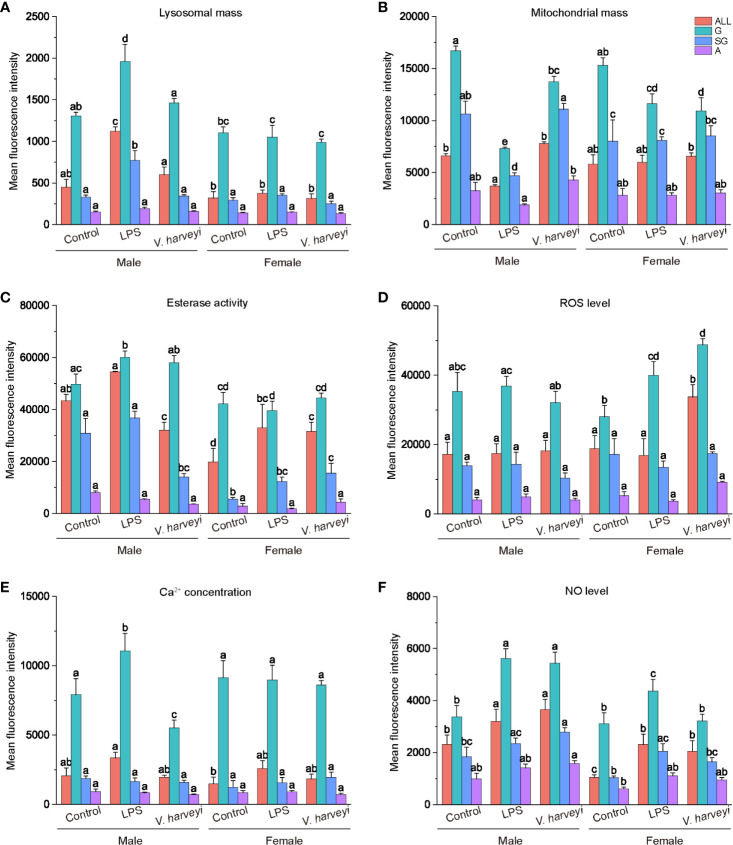
Immunological characteristics of all hemocyte subpopulations after stimulation. **(A)** lysosomal mass, **(B)** mitochondrial mass, **(C)** esterase activity, **(D)** ROS level, **(E)** intracellular calcium concentration, **(F)** NO level. The means denoted by different letters for each fixed hemocyte subpopulation are significantly different among the different stimulations (*p* < 0.05). G, granulocytes; SG, semi-granulocytes; A, agranulocytes.

ROS and NO levels, lysosome and mitochondrial masses, calcium concentrations, and non-specific esterase activity were evaluated using the flow cytometer. The relative mean fluorescence intensities of the granulocytes for the six immunological parameters were significantly higher compared with the corresponding semi-granulocyte and agranulocyte readings under all situations (**Figure 6**).

After immune stimulation with LPS or *V. harveryi*, lysosomal masses in all hemocyte subpopulations were significantly altered by interactions between immune stimulation and gender (**Table S1**). Granulocytes and semi-granulocytes from male oysters exhibited significantly higher lysosomal masses after LPS stimulation (**Figure 6A**).

Mitochondrial masses of total hemocytes, granulocytes, and semi-granulocytes were significantly affected by immune stimulation, gender, and their interactions, at most time points (**Table S1**). The granulocytes of male and female oysters showed significantly lower mitochondrial masses under the two stimulation conditions (**Figure 6B**).

The esterase activity of each hemocyte subpopulation was significantly affected by interactions between immune stimulation and gender (**Table S1**). Esterase activity values in all the hemocytes of female oysters were significantly lower than those of males (**Figure 6C**). Furthermore, both LPS and *V. harveryi* challenge significantly increased the esterase activities of granulocytes from male oysters, whereas those from females exhibited no change.

Granulocyte ROS production levels were significantly affected by immune stimulation and gender (**Table S1**). As shown in **Figure 6D**, immune stimulation did not affect the intracellular ROS concentration of any hemocyte subpopulation in male oysters; whereas LPS and infection by *V. harveryi* significantly increased the ROS concentration of granulocytes in female oysters.

Immune stimulation and the interactions between immune stimulation and gender significantly affected the intracellular calcium levels of granulocytes (**Table 1**). As shown in **Figure 6E**, all hemocyte subpopulation of female oysters showed no significant response in calcium levels to immune stimulations. However, the intracellular calcium levels in male oyster granulocytes were upregulated after LPS stimulation and downregulated after *V. harveryi* challenge.

NO production levels were significantly affected by interactions between immune stimulation and gender (**Table S1**). After immune stimulation, NO production levels of total hemocytes, granulocytes, and semi-granulocytes significantly increased in both male and female oysters. However, the rate of increase in females was lower than that in males.

### Correlation Analysis for Immune Parameters

**Figure 7:**
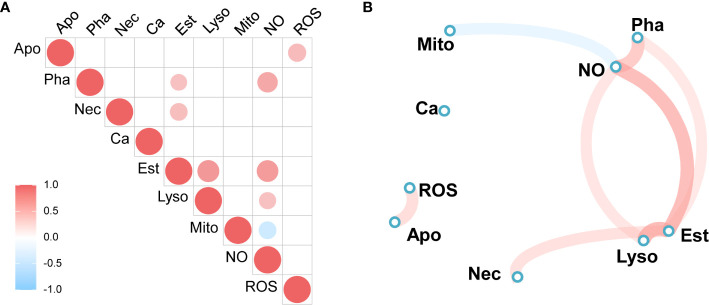
Spearman’s correlation analysis of immune parameters. **(A)** Heatmap of correlation coefficients, **(B)** Correlation network diagram. Red to sky-blue represents positive to negative correlations. Mito, mitochondrial mass; NO, NO level; Ca, calcium content, Pha, phagocytic ratio; Est, esterase activity; ROS, ROS level; Lyso, lysosome mass; Apo, early apoptotic ratio; Nec, late apoptotic or necrotic ratio.

A correlation heatmap and network diagram were applied to represent the Spearman’s correlation coefficients among the immunological parameters of granulocytes, including lysosome and mitochondrial masses; NO, ROS and calcium levels; and phagocytic, early apoptotic, and late apoptotic or necrotic ratios of the total hemocytes. The significant correlations suggest that the parameters were in equilibrium with each other or the concentrations of correlated parameters were simultaneously controlled by the different forms of immune stimulation. As shown in **Figure 7A**, granulocyte NO levels were positively correlated with phagocytic ratio, esterase activities, and lysosome mass, and negatively associated with mitochondrial mass. Esterase activities showed a positive correlation with lysosome mass, late apoptotic or necrotic ratio, and phagocytic ratio. Moreover, in the Spearman’s analysis, NO levels were adjacent to the phagocytic ratios, and esterase activities were close to lysosome masses (**Figure 7B**), indicating biological relationships between them.

## Discussion

The Hong Kong oyster *C. hongkongensis* is one of the most commercially farmed oysters in China. However, the frequent occurrence of infectious diseases in *C. hongkongensis*, especially after spawning, is a major problem in the oyster aquaculture industry. To prevent mortality and subsequent management in Hong Kong oyster farms, an understanding of the oyster immune system is crucial (25). Genetic studies have shown that mollusk hemocytes are essential immune cells with many functions, including phagocytosis, hemolymph clotting, encapsulation, and the production of antimicrobial compounds (22). Hemocytes in mollusks comprise morphologically and functionally diverse subpopulations characterized by different physical properties such as cell size, granularity, and nucleus-cytoplasm ratio (14). In the present study, we used Wright-Giemsa staining and flow cytometry to characterize the hemocyte subpopulations from the Hong Kong oyster *C. hongkongensis*, and agranulocytes, semi-granulocytes, and granulocytes were easily distinguished and separated. Three hemocyte subpopulations have also been identified in other oyster species: the Pacific oyster *C. gigas* (19), the Suminoe oyster *C. ariakensis* (26), and the European flat oyster *Ostrea edulis* (27). Li et al. (25) reported that the circulating hemocytes of *C. hongkongensis* could be separated into hyalinocytes and granulocytes. It is noteworthy that the osmolality of the anticoagulant used in that study was approximately 1000 mOsm/kg, which is much higher than the normal osmotic pressure in Hong Kong oysters (< 650 mOsm/kg). The high osmotic pressure may have caused cell morphology changes, and thus the hyalinocytes were suspected to be composed of semi-granulocytes and agranulocytes. Cell sorting combined with the transcriptome analysis of *C. hongkongensis* hemocytes also indicated that the semi-granulocytes and agranulocytes were two different populations (Lu et al., unpublished).

In the present study, we discovered that lysosome and mitochondrial masses, NO and ROS levels, and phagocytic and non-specific esterase activities were mainly concentrated in granulocytes under all conditions. Granulocytes were reported to be the main immunocompetent hemocytes in *C. gigas* (19), *Pila globose*, and *Lamellidens marginalis* (28); therefore, we speculated that granulocytes are also the principal immune hemocytes in *C. hongkongensis*. Many studies have indicated that the immunological parameters of mollusk hemocytes show some variations in response to different immune stimulations (2, 4). However, only a few studies have highlighted the influence of gender on immune functions in marine mollusks; therefore, we used multivariate statistical methods to evaluate the effects of immune stimulation and gender on immunological parameters. Both the MANOVA and PCA results showed that both immune stimulation and gender affected the hemocyte immune parameters in *C. hongkongensis*, and an interaction effect was also evident. However, almost no differences in the hemocyte immune parameters of male and female oysters under normal conditions were found. To our knowledge, this is the first report describing gender-related differences in the immunological parameters of the Hong Kong oyster *C. hongkongensis* after immune stimulation.

THC is an essential immunological parameter for predicting the health of mollusks because hemocytes migrate from the circulatory system to tissues to help resist invading pathogens. This study showed that the hemolymph of *C. hongkongensis* had a hemocyte concentration of 1.12 ± 1.1 × 10^6^ cells/mL, which was lower than the concentration measured by Li et al. (25) (2.52 ± 1.1 × 10^6^ cells/mL). Previous studies have shown that hemocyte concentrations can be affected by endogenous (e.g., age, size, gender, and reproductive period) and exogenous (e.g., temperature, salinity, pH, and pollutants) factors (29, 30). It is likely the differences in the total number of hemocytes seen in the present study and that by Li et al. (25) were due to size, reproductive period, or sampling season. In agreement with previous studies (31, 32), two immune stimulation types led to a decrease in the THC, but no significant difference in the THC was found between genders. Similarly, Cheng et al. (33) also reported no significant difference in the THC between genders of *Macrobrachium rosenbergii*, and Duchemin et al. (5) observed that THC did not differ with gender in triploid or diploid *C. gigas*. Reportedly, the percentages of the different cell subpopulations in the hemolymph can vary according to environmental and pathogenic stimulation (2, 34). We found no significant differences in the numbers of hemocyte subpopulations between male and female oysters under normal conditions in this study. However, two immune stimulations induced increases in the agranulocyte populations of male oysters and decreases in those of females. Conversely, a higher proportion of active granulocytes was observed in female *Ruditapes philippinarum* clams (4). This difference may be attributed to the different reproductive states of the animals, as Matozzo and Marin (4) sampled clams during the pre-spawning phase, whereas the population in the present study was collected after spawning. Because granulocytes were the main immunocompetent hemocytes, the increased proportion of granulocytes in males shows that the males had more active hemocytes than the females under immune-activated situations. These findings demonstrate that immune stimulation induced the gender-specific stress responses in hemocyte subpopulations.

Annexin-V assays, which are reliably used to detect apoptotic and necrotic cells in mammals, were used to quantify the innate defense mechanism of *C. hongkongensis* by adjusting the reagent osmolalities to 600 mOsm/kg. This work demonstrated the high percentages of late apoptotic or necrotic cells in total hemocytes from male and female oysters after immune stimulation. A significant inverse correlation (r = −0.85, *p* < 0.05) was observed between the number of total hemocytes and the percentage of late apoptotic or necrotic cells. Similar to previous findings (31), the phenomenon revealed that the disappearance of the hemocytes correlated with cell necrosis and apoptosis. Moreover, as previously observed in *C. gigas* (5), no gender difference in late apoptosis and necrosis of hemocytes was observed in *C. hongkongensis*. Apoptosis, an orchestrated physiological process of cellular self-destruction, is essential for the correct functioning of the molluscan immune system (35). We observed lower early apoptosis rates for all granulocytes and higher apoptosis rates for semi-granulocytes in female oysters compared with males after stimulation by *V. harveyi* infection, suggesting that the semi-granulocytes in females were more susceptible to *V. harveyi* infection. The release of ROS by hemocytes is a key internal defense mechanism by which pathogens are destroyed before their phagocytosis (36). Higher ROS production was detected in female than male granulocytes, especially after LPS or *V. harveryi* stimulation. Gender-dependent differences were also reported in the abundance of ROS in the hemocytes of *Saccostrea glomerata* and *Pinctada fucata* (2). As shown in **Figure 7**, ROS was positively correlated with early apoptosis. Excess cellular levels of ROS have been shown to induce apoptosis (37). The higher levels of ROS, combined with the higher early apoptosis rate, show that *V. harveryi* challenge induced the apoptosis of female hemocytes *via* ROS generation. These findings indicate that male and female oysters use different intracellular oxidative metabolic strategies to resist LPS or *V. harveyi* infection.

Phagocytosis is an essential and effective defense mechanism against foreign pathogens. A decrease in the male and female oyster phagocytic index was witnessed in the present study, from strong phagocytosis in granulocytes, weak phagocytosis in semigranulocytes, to no phagocytosis in agranulocytes. Similar results have been reported for *C. gigas* (19). The phagocytic ratio of the total granulocytes was significantly upregulated after LPS challenge, but no significant difference was detected after *V. harveyi* challenge. Jiang et al. (38) reported that LPS, but not peptidylglycan, significantly increased phagocytic activities in *C. gigas*. These results indicated that different stimulants induced phagocytic activities *via* different strategies. Furthermore, statistical analysis revealed that male oysters had slightly (although not significantly) more phagocytic ratios than females under all corresponding conditions. Female and male diploid *C. gigas* also showed no statistically significant differences in their phagocytic index (5). NO has many biological functions related to defense and immune responses in marine invertebrates (28). In the present study, both LPS and *V. harveryi* stimulation induced a noticeable gender-specific increase in NO levels. Thus, in hemocytes, NO appears to play a pivotal role in the killing of intracellular pathogens. NO was also shown to be involved in defense mechanisms in the mollusk *Mytilus edulis* (39), and it appeared to be a cellular signal involved in the response to environmental stress in *C. virginica* (40). The NO produced by the immune cells of Wistar rats had a role in intracellular killing and phagocytic activity (41). Coincidentally, correlation analysis showed that granulocyte NO levels significantly correlated with the phagocytic ratios (**Figure 7**). Therefore, we concluded that the hemocytes of *C. hongkongensis* generate NO as a cytokine to regulate the phagocytic activities protecting the hosts from LPS or *V. harveyi* infection. Additionally, the significantly higher NO levels and the non-significant higher phagocytic ratio of males oysters after immune stimulation also indicate that males are more immunocompetent than females. Mitochondria, responsible for the energy production processes necessary for cell metabolism, vary in their number, activity, and localization in animal cells in relation to energetic needs (42). A decrease in mitochondrial function is often accompanied by an increase in proton leak, inhibition of vital mitochondrial enzymes, and elevated production of nitric oxide and reactive oxygen species (43). Notably, the mitochondrial mass showed a negative correlation with the level of nitric oxide (**Figure 7**); therefore, the lower mitochondrial mass observed in both male and female oysters after immune stimulation might be due to a higher concentration of NO. However, this needs further research.

Lysosomes, which are important bacteriolytic cellular organelles, are employed as an index to evaluate the health status and vitality of the defense system in bivalves (29) and are generally affected by environmental stress (30). Lysosomal masses of *C. gigas* granulocytes were significantly increased after stimulation with *V. splendidus* (19). Similar results were found for the male *C. hongkongensis* in this study following LPS stimulation, indicating gender-specific lysosomal responses by granulocytes to LPS stimulation. The gender-dependent differences in lysosomal masses were also reported for *Ruditapes philippinarum* (4) and *Panorpa vulgaris* (44); however, it was the hemocytes of the females of these two species that showed higher lysosomal masses. Intracellular calcium not only participates in various biological activities, such as metabolism regulation and biomineralization, but also acts as a ubiquitous second messenger to regulate intracellular or intercellular signal transduction (45, 46). The stress of organelles, including the endoplasmic reticulum, mitochondria, and lysosomes, might lead to the release of calcium into the cytoplasm (47). The concentrations of intracellular calcium in male granulocytes increased after LPS treatment, suggesting that intracellular calcium served as an essential mediator in the immune response, and much more calcium was required to maintain the lysosome mass. This speculation is supported by the higher lysosome mass in male granulocytes under LPS challenge. Increased intracellular calcium levels and lysosome masses were also observed in P1 hemocytes of *Eriocheir sinensis* (14). The hydrolase enzyme non-specific esterase plays a pivotal role in intracellular degradation and the stress response in the hemocytes of mussels (30, 48). In the present study, we observed gender differences in the esterase activities of male and female oysters, with lower levels in female oysters compared with males under all conditions. Higher hydrolytic enzyme activity has also been observed in male *R. philippinarum* compared with females. In *C. virginica*, non-specific esterase was detected and inferred to be associated with lysosome-like bodies (49); interestingly, there were significant positive correlations between the esterase activity and lysosomal mass. The esterase activity in *C. hongkongensis* granulocytes was adjacent to lysosomal mass in the Spearman’s correlation analysis. Hence, the gender-specific activity of esterase could be considered a consequence of gender-specific differences in lysosomal mass. The higher esterase activities and lysosomal masses in males compared with females further suggest that gender-specific immune responses were induced in *C. hongkongensis* hemocytes.

Generally, adult females mount stronger innate and adaptive immune responses than males (50). Many theories, such as the immunocompetence handicap hypothesis (ICHH) (51), Bateman’s principle (52), evolutionary-ecology approach (53), and the sicker sex principle (54), attempt to explain why gender differences exist. However, in this study, the upregulation of granulocyte esterase activities, lysosomal masses, nitric oxide levels, and granulocyte numbers was observed in male *C. hongkongensis.* These findings indicate that males have a more powerful cellular immune response level than females after spawning. Because Hong Kong Oysters reproduce using external fertilization, we speculate that females may invest more reproductive resources and have a weaker immune system after spawning. This speculation can be proved by the high mortality of post-spawning-phase female Hong Kong oysters. In the sea cucumber *A. japonicus*, the stronger antioxidant ability is also observed in males than that in females after spawning (6). In the current study, we have analyzed the differences in immunity to infections between male and female oysters based on hemocyte immune parameters, but humoral immunity systems such as the phenoloxidase system (55) also play an important role in molluscan immunity, which is worthy of further study.

## Conclusion

In this study, gender-related differences in immune responses to LPS and *V. harveyi* were reported for the first time in the Hong Kong oyster *C. hongkongensis* during the post-spawning phase. To accurately assess the immune parameters in hemocytes, three types of hemocyte were identified: granulocytes, semi-granulocytes, and agranulocytes. Because granulocytes were identified as the primary phagocytes, with a dense mass of mitochondria and lysosomes and prominent esterase, superoxide anion, and nitric oxide activities, we concluded that granulocytes are the main immunocompetent hemocytes in *C. hongkongensis*. Our multivariate statistical results showed that gender, immune stimulation, and their interaction, affected the immune-related parameters of hemocyte subpopulations. Significantly lower THC values were recorded in both male and female oysters, but significantly higher percentages of granulocytes were found in the hemolymph of males after immune stimulation compared with that of females. Esterase activities and lysosomal masses were positively correlated, and they significantly increased in male hemocytes after immune challenge. NO levels were also upregulated in males and were positively associated with non-significantly higher phagocytic ratios in males post-immune infection. These results suggest that, during the post-spawning stage, male oysters have more effective defense responses against immune infection than females. Therefore, gender and subpopulation differences should be included in the future analysis of bivalve immune parameters when studying the impact of pathogens, environmental variables, or multiple variables.

## Data Availability Statement

The original contributions presented in the study are included in the article/**Supplementary Material**. Further inquiries can be directed to the corresponding authors.

## Ethics Statement

The animal study was reviewed and approved by The Animal Care and Ethics Committee of South China Sea Fisheries Research Institute, Chinese Academy of Fishery Sciences.

## Author Contributions

JL: funding acquisition, methodology, validation, data curation, writing—original draft, writing—review & editing. YS: methodology, writing—review and editing. TY: Writing—review and editing. CB: Writing—review and editing. JJ: writing—review and editing. LY: funding acquisition, writing—review and editing. All authors contributed to the article and approved the submitted version.

## Funding

This work was financially supported by National Key R&D Program of China (2019YFD0900105), Central Public-interest Scientific Institution Basal Research Fund, South China Sea Fisheries Research Institute, CAFS (2017YB22), the earmarked fund for Modern Agro-industry Technology Research System (CARS-49), the Central Public-interest Scientific Institution Basal Research Fund, CAFS (2020TD42, 2021SD05), National Key R&D Program of China (2019YFD0900105), the Science and Technology Planning Project of Guangzhou (202002030488), the Shellfish and Large Algae Industry Innovation Team Project of Guangdong Province (2021KJ146), the Professorial and Doctoral Scientific Research Foundation of Huizhou University (2020JB0650), and Provincial Rural Revitalization Strategy Special Funds (Fishery Industry Development).

## Conflict of Interest

The authors declare that the research was conducted in the absence of any commercial or financial relationships that could be construed as a potential conflict of interest.
