# Supplementary material for: Gender Differences in Hemocyte Immune Parameters of Hong Kong Oyster Crassostrea hongkongensis During Immune Stress
Source: Front Immunol. 2021 Mar 31;12:659469. doi: 10.3389/fimmu.2021.659469 (PMC8044396; doi:10.3389/fimmu.2021.659469)
Supplement: Supplementary file 1 [file Table_1.docx]

**Gender differences in hemocyte immune parameters of Hong Kong oyster *Crassostrea hongkongensis* during immune stress**

Jie Lu ^a *^, Yanyan Shi ^b^, Tuo Yao ^a^, Jiangyong Wang ^c^, Lingtong Ye ^a *^

^a^ *Key Laboratory of South China Sea Fishery Resources Exploitation & Utilization, Ministry of Agriculture, South China Sea Fisheries Research Institute, Chinese Academy of Fishery Sciences, Guangzhou 510300, P.R. China*

*^b^ Department of Chemical and Biochemical Engineering, College of Chemistry and Chemical Engineering, Xiamen University, Xiamen 361005, P.R. China*

*^c^ Huizhou University, Huizhou 516007, P.R. China*

* Corresponding authors at: South China Sea Fisheries Research Institute, Chinese Academy of Fishery Sciences, Guangzhou 510300, P.R. China. E-mail address: [lujie@scsfri.ac.cn](mailto:lujie@scsfri.ac.cn), lingtong2753@126.com.

**Table S1.** Summary of two-way ANOVA results on effects of gender and stimulation on hemocyte counts (HC), mitochondrial mass (Mito), NO level (NO), esterase activity (Est), ROS level (ROS), lysosome content (Lyso) and calcium content (Ca) early apoptotic ratio (Apo), late apoptotic or necrotic ratio (Nec) in all hemocytes (ALL), granulocytes (G), semi-granulocytes (SG), and agranulocytes (A) from *Crassostrea hongkongensis.*

|  | Gender | | | | Stimulation | | | | Sex*Stimulation | | | |
| --- | --- | --- | --- | --- | --- | --- | --- | --- | --- | --- | --- | --- |
|  | df | MS | F | P | df | MS | F | P | df | MS | F | P |
| HC_ALL | 1 | 0.001 | 0.390 | 0.538 | 2 | 0.006 | 2.632 | 0.042 | 2 | 0.002 | 0.665 | 0.523 |
| HC_G | 1 | 0.322 | 30.537 | 0.000 | 2 | 0.052 | 4.968 | 0.015 | 2 | 0.184 | 17.449 | 0.000 |
| HC_SG | 1 | 0.090 | 7.651 | 0.011 | 2 | 0.131 | 11.092 | 0.000 | 2 | 0.187 | 15.781 | 0.000 |
| HC_A | 1 | 0.318 | 36.177 | 0.000 | 2 | 0.246 | 27.991 | 0.000 | 2 | 0.055 | 6.226 | 0.006 |
| Apo_ALL | 1 | 0.006 | 26.787 | <0.001 | 2 | 0.010 | 42.458 | <0.001 | 2 | 0.010 | 41.149 | <0.001 |
| Pha_ALL | 1 | 0.002 | 0.730 | 0.401 | 2 | 0.001 | 0.463 | 0.635 | 2 | 0.011 | 4.494 | 0.022 |
| Nec_ALL | 1 | 0.001 | 1.685 | 0.206 | 2 | 0.004 | 5.657 | 0.009 | 2 | <0.001 | 0.805 | 0.458 |
| Ca_ALL | 1 | 0.007 | 0.132 | 0.720 | 2 | 0.088 | 1.558 | 0.230 | 2 | 0.183 | 3.246 | 0.056 |
| Est_ALL | 1 | 0.064 | 2.560 | 0.122 | 2 | 0.066 | 2.657 | 0.090 | 2 | 0.197 | 7.926 | 0.002 |
| Lyso_ALL | 1 | 0.090 | 2.959 | 0.098 | 2 | 0.089 | 2.910 | 0.073 | 2 | 0.526 | 17.224 | <0.001 |
| Mito_ALL | 1 | 0.067 | 18.058 | <0.001 | 2 | 0.038 | 10.313 | <0.001 | 2 | 0.076 | 20.371 | <0.001 |
| NO_ALL | 1 | 0.068 | 2.817 | 0.106 | 2 | 0.047 | 1.965 | 0.161 | 2 | 0.402 | 16.672 | <0.001 |
| ROS_ALL | 1 | 0.040 | 0.790 | 0.382 | 2 | 0.087 | 1.701 | 0.203 | 2 | 0.017 | 0.343 | 0.713 |
| Apo_G | 1 | <0.001 | 1.745 | 0.198 | 2 | <0.001 | 1.732 | 0.198 | 2 | <0.001 | 5.114 | 0.014 |
| Pha_G | 1 | <0.001 | 0.071 | 0.792 | 2 | 0.002 | 0.905 | 0.417 | 2 | 0.007 | 4.053 | 0.030 |
| Nec_G | 1 | <0.001 | 1.069 | 0.311 | 2 | <0.001 | 2.548 | 0.098 | 2 | <0.001 | 4.801 | 0.017 |
| Ca_G | 1 | 0.055 | 3.959 | 0.058 | 2 | 0.056 | 4.016 | 0.031 | 2 | 0.049 | 3.513 | 0.045 |
| Est_G | 1 | 0.011 | 3.393 | 0.077 | 2 | 0.013 | 4.130 | 0.028 | 2 | 0.057 | 17.679 | <0.001 |
| Lyso_G | 1 | 0.060 | 12.203 | 0.002 | 2 | 0.017 | 3.383 | 0.050 | 2 | 0.109 | 22.104 | <0.001 |
| Mito_G | 1 | 0.006 | 1.687 | 0.206 | 2 | 0.141 | 38.493 | <0.001 | 2 | 0.079 | 21.480 | <0.001 |
| NO_G | 1 | 0.029 | 2.620 | 0.118 | 2 | 0.010 | 0.916 | 0.413 | 2 | 0.150 | 13.677 | <0.001 |
| ROS_G | 1 | 0.063 | 6.543 | 0.017 | 2 | 0.074 | 7.762 | 0.002 | 2 | 0.010 | 1.033 | 0.371 |
| Apo_SG | 1 | 0.006 | 50.041 | <0.001 | 2 | 0.006 | 46.636 | <0.001 | 2 | 0.005 | 41.051 | <0.001 |
| Pha_SG | 1 | <0.001 | 2.227 | 0.148 | 2 | <0.001 | 0.687 | 0.513 | 2 | <0.001 | 1.564 | 0.229 |
| Nec_SG | 1 | <0.001 | 0.086 | 0.771 | 2 | 0.002 | 11.492 | <0.001 | 2 | <0.001 | 1.246 | 0.305 |
| Ca_SG | 1 | 0.026 | 0.469 | 0.500 | 2 | 0.067 | 1.200 | 0.318 | 2 | 0.092 | 1.640 | 0.214 |
| Est_SG | 1 | 0.162 | 2.003 | 0.169 | 2 | 0.243 | 2.991 | 0.068 | 2 | 0.718 | 8.847 | 0.001 |
| Lyso_SG | 1 | 0.117 | 8.067 | 0.009 | 2 | 0.042 | 2.919 | 0.073 | 2 | 0.256 | 17.703 | <0.001 |
| Mito_SG | 1 | 0.050 | 3.791 | 0.063 | 2 | 0.110 | 8.278 | 0.002 | 2 | 0.137 | 10.341 | <0.001 |
| NO_SG | 1 | 0.084 | 5.655 | 0.025 | 2 | 0.022 | 1.498 | 0.243 | 2 | 0.233 | 15.707 | <0.001 |
| ROS_SG | 1 | 0.020 | 1.206 | 0.283 | 2 | 0.026 | 1.538 | 0.234 | 2 | 0.047 | 2.815 | 0.079 |
| Apo_A | 1 | <0.001 | 0.299 | 0.590 | 2 | <0.001 | 5.874 | 0.008 | 2 | 0.002 | 15.009 | <0.001 |
| Nec_A | 1 | 0.001 | 4.128 | 0.053 | 2 | <0.001 | 1.694 | 0.204 | 2 | <0.001 | 0.938 | 0.405 |
| Ca_A | 1 | 0.007 | 0.291 | 0.594 | 2 | 0.009 | 0.352 | 0.707 | 2 | 0.009 | 0.374 | 0.692 |
| Est_A | 1 | 0.224 | 7.756 | 0.010 | 2 | 0.146 | 5.041 | 0.014 | 2 | 0.411 | 14.229 | <0.001 |
| Lyso_A | 1 | 0.017 | 2.701 | 0.113 | 2 | <0.001 | 0.100 | 0.905 | 2 | 0.037 | 6.069 | 0.007 |
| Mito_A | 1 | 0.136 | 7.222 | 0.013 | 2 | 0.052 | 2.753 | 0.083 | 2 | 0.053 | 2.790 | 0.081 |
| NO_A | 1 | 0.061 | 3.976 | 0.057 | 2 | 0.035 | 2.270 | 0.124 | 2 | 0.223 | 14.626 | <0.001 |
| ROS_A | 1 | 0.030 | 1.269 | 0.271 | 2 | 0.177 | 7.516 | 0.003 | 2 | 0.131 | 5.574 | 0.010 |
